# I Don’t Want to Miss a Thing – Learning Dynamics and Effects of Feedback Type and Monetary Incentive in a Paired Associate Deterministic Learning Task

**DOI:** 10.3389/fpsyg.2017.00935

**Published:** 2017-06-08

**Authors:** Magda Gawlowska, Ewa Beldzik, Aleksandra Domagalik, Adam Gagol, Tadeusz Marek, Justyna Mojsa-Kaja

**Affiliations:** ^1^Department of Forensic Psychology, Institute of Applied Psychology, Jagiellonian UniversityKrakow, Poland; ^2^Neurobiology Department, Malopolska Centre of Biotechnology, Jagiellonian UniversityKrakow, Poland; ^3^Department of Cognitive Neuroscience and Neuroergonomics, Institute of Applied Psychology, Jagiellonian UniversityKrakow, Poland; ^4^Neurocognitive Processing Laboratory, Institute of Philosophy, Jagiellonian UniversityKrakow, Poland; ^5^Department of Neurobiology and Neuropsychology, Institute of Applied Psychology, Jagiellonian UniversityKrakow, Poland

**Keywords:** learning dynamics, learning curve, deterministic learning, decision-making, motivation

## Abstract

Effective functioning in a complex environment requires adjusting of behavior according to changing situational demands. To do so, organisms must learn new, more adaptive behaviors by extracting the necessary information from externally provided feedback. Not surprisingly, feedback-guided learning has been extensively studied using multiple research paradigms. The purpose of the present study was to test the newly designed Paired Associate Deterministic Learning task (PADL), in which participants were presented with either positive or negative deterministic feedback. Moreover, we manipulated the level of motivation in the learning process by comparing blocks with strictly cognitive, informative feedback to blocks where participants were additionally motivated by anticipated monetary reward or loss. Our results proved the PADL to be a useful tool not only for studying the learning process in a deterministic environment, but also, due to the varying task conditions, for assessing differences in learning patterns. Particularly, we show that the learning process itself is influenced by manipulating both the type of feedback information and the motivational significance associated with the expected monetary reward.

## Introduction

Functioning in a complex and unpredictable environment forces organism to adjust behavior according to changing situational demands. It is crucial to learn new, more adaptive behaviors in accordance with external feedback and to test whether these new behaviors lead to the expected, more efficient outcomes. Not surprisingly, feedback-guided learning has become an extensively studied topic over decades of experimental research (De Houwer, 2009; Chase et al., 2011; De Houwer et al., 2013).

There are various paradigms used in experimental research to assess the process of learning. In general, they can be classified into two main categories based on the character of the environment that learning takes place in: paradigms operating on uncertain (probabilistic) feedback, and those operating on certain (deterministic) feedback. In probabilistic learning paradigms, it is harder for participants to achieve perfect performance due to the fact that feedback information does not always match a given response. On the contrary, in deterministic learning the response to certain stimulus is associated with only correct or incorrect feedback, but not both (the probability of a certain outcome equals 1). Besides this obvious distinction, learning in probabilistic and deterministic conditions differs in other ways. Probabilistic tasks are perceived as more difficult, because the feedback information is not always valid (Mehta and Williams, 2002; Juslin et al., 2003). The dynamics of learning have been extensively studied using probabilistic learning paradigms (e.g., Frank et al., 2005, 2007a; Krigolson and Holroyd, 2006; Holroyd and Krigolson, 2007; Frank and Kong, 2008) because they are believed to better simulate the decision process and probability of a favorable outcome in every-day life (van de Vijver et al., 2014). However, as probabilistic learning is considered more difficult, not every participant is able to comply with task demands. For example, older adults or amnesic patients are known to present greater impairment in probabilistic than deterministic learning (Vandenberghe et al., 2006; Eppinger et al., 2008; van de Vijver et al., 2014). To identify the optimal response, a participant has to integrate the outcomes of multiple trials featuring the same stimulus; therefore, probabilistic learning paradigms are associated with higher cognitive demands (Reed et al., 2014), whereas deterministic learning seems to mirror the pure contingency learning process (Vanes et al., 2014).

Behavioral studies on learning with cognitive feedback suggest that tasks in which subjects have to rely on gradually acquired stimulus–outcome contingencies are sensitive to both the type of presented feedback and its temporal proximity (Maddox et al., 2003). Common sense implies the more feedback information during learning, the better, as it provides additional details that can be used when adapting more efficient learning strategies (Salmoni et al., 1984). However, as feedback frequency increases, individuals must respond to and process more information which, in turn, consumes more of the available cognitive resources. When provided feedback is too frequent, it can impair an individual’s ability to learn (Lam et al., 2011; Mohammadi et al., 2011). A common strategy in most learning paradigms is to follow each response with feedback; however, as showed by Lam et al. (2011), it may be more beneficial for the learning process to decrease the overall feedback amount.

The aforementioned difficulties regarding experimental paradigms designed to study the learning process prompt one to ask whether using simpler learning tasks involving reduced, deterministic feedback would allow all the necessary information about learning dynamics to be collected. Thus, in this paper, we present a new experimental paradigm that is designed to study the learning process in a simple, less demanding deterministic environment: The Paired Associate Deterministic Learning task (PADL). Moreover, using the PADL we investigate if participants presented with a reduced amount of deterministic feedback are able to master a task sufficiently.

Finally, the learning process may be influenced not only by the difficulty of the task or the cognitive overload, but also by the motivational salience participants ascribe to their performance and to the consequences of their performance. As shown by Kahneman and Tversky, people tend to be more sensitive to the possibility of losing than they are to the possibility of gaining objects or money (Tversky and Kahneman, 1992). This trend of loss aversion is seen both in young children and primates (e.g., capuchin monkeys), thus suggesting its evolutionary basis (Tom et al., 2007). However, most studies in humans have used monetary rewards and punishments as feedback and it is not entirely clear whether monetary incentives are qualitatively different from non-monetary performance feedback (e.g., ‘correct’ or ‘incorrect’) (Wheeler and Fellows, 2008). Some conclusions can be drawn from neuroimaging studies which show the activation of different brain structures during processing of positive and negative feedback. The rostral cingulate zone (RCZ), associated with transmission of a prediction error (PE) signal by the mesencephalic dopaminergic system, is more active during the processing of negative feedback, whereas the nucleus accumbens (NAcc) shows greater activity during the processing of positive feedback (Daniel and Pollmann, 2010). Moreover, the anticipation of monetary reward reflects in higher activation in the NAcc, compared to the anticipation of cognitive feedback. Activation in the NAcc has been shown to increase with both reward magnitude and reward probability (Knutson et al., 2001; Abler et al., 2006). These results, although they cannot be transferred to the behavioral level, encourage the search for possible differences.

Taken together, the aim of our study was to test whether the PADL, an experimental paradigm operating on the rules of deterministic environment with feedback information limited to either positive or negative, is suitable to provide data describing the dynamics of the deterministic learning process. We extend the presented results by comparing blocks with strictly cognitive, informative feedback that refers only to the correctness of an answer (“good” or “bad”) with blocks in which participants were additionally motivated by anticipated monetary reward or loss. Thus, we hypothesize that the PADL can be considered a useful experimental task for analyzing both the dynamics of a deterministic learning process and the accompanying differences due to the motivational significance of the experimental conditions. In particular: 1) it allows the differences in learning dynamics to be investigated due to the type of presented feedback and 2) it allows the differences in learning dynamics to be investigated, depending on the presence of a monetary incentive.

## Materials and Methods

### Participants

The study was conducted on a group of 62 participants (mean age: 23, *SD* = 2.3, 32 females). Participation was voluntary and each subject was paid for taking part in the experiment. All the participants were right-handed Caucasians without any history of neurological disorders. Participants were informed about the procedure and goals of the study and gave their written consent. The data analyses were performed on 58 participants. The four participants were excluded due to failure to comply with the task instruction. The study was approved by the Bioethics Commission at Jagiellonian University and all subjects gave written informed consent in accordance with the Declaration of Helsinki.

### Task

**FIGURE 1:**
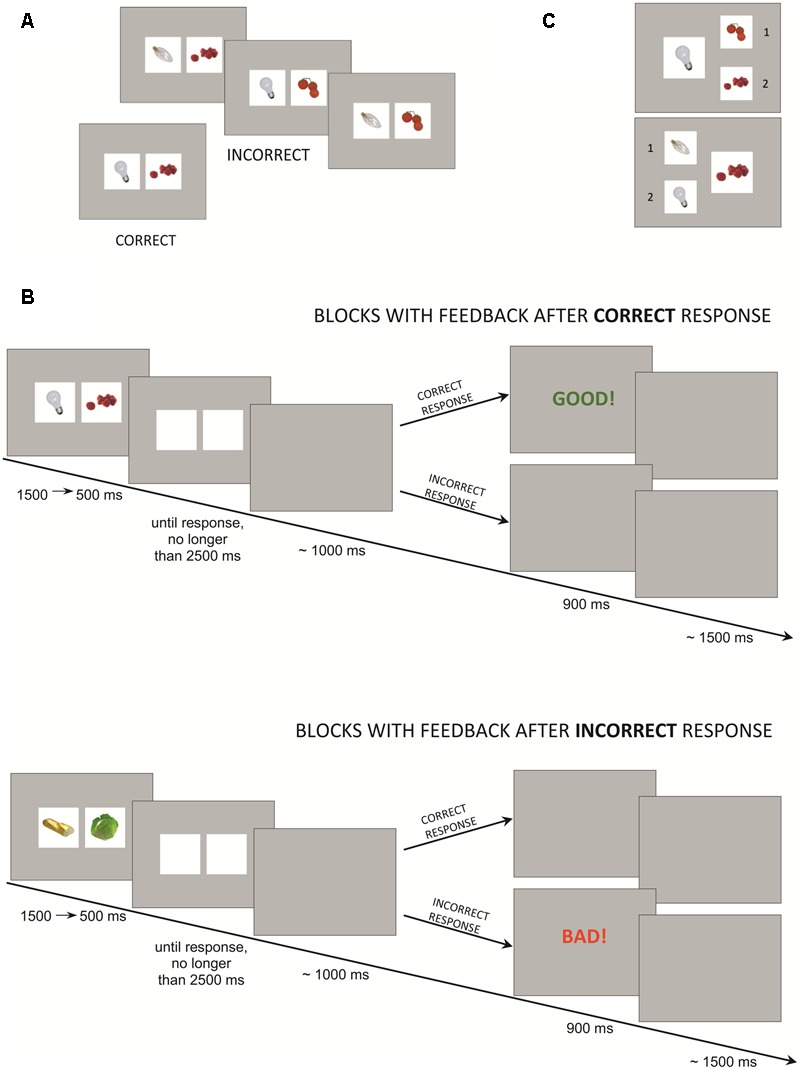
Experimental task: **(A)** Sample stimuli set from the learning block. **(B)** Task design. **(C)** Sample pair of stimuli from the test phase.

The task consisted of four learning blocks, each followed by a test phase. In the learning blocks, participants were presented with pairs of pictures and asked to learn based on the presented feedback whether the pair was correct or not and to submit the answer by pressing 1 (“correct”) or 2 (“incorrect”) on a keypad. In every block, there were nine unique stimuli sets. Each set comprised one correct pair of pictures and three distracting pairs that were similar to the correct one (**Figure 1A**). The similarity was established based on the shape, color or category appurtenance, e.g., if the correct pair was a round, white light bulb and raspberries, the distracting pairs were: a round, white light bulb and cherry tomatoes; a transparent, elongated light bulb and raspberries; or a transparent, elongated light bulb and cherry tomatoes. Thirty-six stimuli (nine sets × four stimuli in every set) were presented five times each: a total of 180 stimuli per block. The stimuli were designed using pictures from BOSS, the Bank of Standardized Stimuli (Brodeur et al., 2010). As the stimuli order was semi-randomized, it allowed five time-points of the learning process to be distinguished: the first time-point was marked by a single presentation of every correct pair and its variations (36 pairs in total), the second time-point was marked by the second presentation, etc. Every subsequent presentation of the stimuli was faster than the previous one: first presentation – 1500 ms, second presentation – 1250 ms, third presentation – 1000 ms, fourth presentation – 750 ms, and fifth presentation – 500 ms. After the stimulus, a blank screen was displayed until 800 to 1200 ms (mean: 1000 ms) after the response was submitted. The response time limit was set to 4000 ms for the 1st presentation of stimuli and gradually decreased by 250 ms in subsequent blocks to 3000 ms for the 5th presentation. A feedback screen displayed for 900 ms was followed by a blank screen for the next 1000 to 2000 ms (mean: 1500 ms), thus accounting for the inter-trial interval (ITI). See **Figure 1B**.

Participants were informed that after each learning block they would take part in the test phase to assess how well they had learned the correct pairs. The test phase consisted of 18 boards (**Figure 1C**). Each board depicted one half of the correct pair (one picture) to which participants had to match the second half from two possible options (picture 1 or picture 2). Pictures were presented in semi-randomized order: the first and the second half of boards (i.e., nine boards) included all the correct pairs. There was no time-limit to submit the response.

The four learning blocks differed in terms of feedback type. In the “GOOD” block participants received feedback only after correct responses; in the “BAD” block participants received feedback only after the incorrect responses. In the “WIN” block participants received feedback after correct responses and were additionally informed that they would be able to win money in the test phase (up to 15 PLN, ∼4$) if they matched pictures correctly. However, in the “LOSE” block participants received feedback only after incorrect responses. Moreover, they were informed they had been given the amount of 15 PLN and they might lose it all if they matched pictures incorrectly in the test phase. Every block was preceded by sufficient information about the feedback and possible reward. In both the “GOOD” and the “WIN” block, subjects were presented with a green “Good!” screen, while in the “BAD” and the “LOSE” block they were presented with a red “Bad!” screen (**Figure 1B**). If a participant exceeded the response time limit, a “too slow” feedback screen was displayed. Block order was semi-counterbalanced across participants: half of the participants started with a “GOOD” block and half with a “BAD” block. However, the block without monetary incentive always preceded the block with money, i.e., the “GOOD” block preceded the “WIN” block and the “BAD” block preceded the “LOSE” block. The full randomization scheme was not applied due to the expected motivational effect of the money condition and to avoid a situation in which participants would not engage in the learning process during blocks without monetary reward.

### Experimental Procedure

The experimental task was prepared and generated using E-Prime 2.0 (©Psychology Software Tools). Stimuli were presented on a 17″ LCD monitor and participants responded by pressing keys 1 or 2 on the Serial Response Box (©Psychology Software Tools) with the left and right index finger, respectively. The day before the main experiment, all participants took part in a training session to get familiarized with the task demands. Subjects were presented with a brief description of the experiment and were given a printed version of the task instruction. Finally, participants performed a training version of the task procedure. At the beginning of the main experiment, participants were again presented with the task instruction. Afterward, they performed the experimental task, which took approximately 40 min.

### Data Analysis

The effect of learning, i.e., the number of learned correct pairs, was assessed by separately computing the percentage of correct responses in the test phase for the four learning conditions.

To examine the learning dynamics, the behavioral data were divided into five learning time-points of 36 trials each, i.e., the first learning time-point contained trials 1–36, the second contained trials 37–72, and so forth. Within each learning time-point, mean reaction times (RTs) were computed separately for the four learning conditions. Further, following the measure implemented by Toni et al. (2001), we analyzed the change in RT variability (vRT) dynamics, i.e., the change in the standard deviation of RTs across the learning time-points and experimental conditions.

As a direct measure of learning, performance discriminability index (D-prime, Signal Detection Theory, see: Macmillan and Creelman, 1990) values were computed for all learning time-points and conditions. Applying D-prime to measure performance accuracy allows not only situations in which a participant learns to choose correctly target stimuli to be considered, but also the situations in which distractors are properly rejected. As a model of task learning, an exponential curve was chosen since it fits better than the power function in most data sets depicting the dynamics of knowledge acquisition (Heathcote et al., 2000) and is equivalent to the sigmoid model for tasks in which correct and erroneous responses have the same potential to affect learning (Leibowitz et al., 2010). In the PADL, the actual amount of feedback information in all the learning blocks was the same since the lack of negative feedback can be interpreted as a sign of a correct response (the converse is also true for positive feedback and erroneous response); therefore, the assumption about the comparable influence of positive and negative feedback on the learning process holds in the case of the PADL. The exact form of the learning curve model is:

(1) $$P_{n}\;=\;P_{\infty }-(P_{\infty }-P_{0})e^{-An}$$

where *P*_n_ denotes performance measure in the *n*-th learning time-point, *P*_0_ and *P*_∞_ denote the initial and asymptotic performance, respectively, and *A* is a constant rate coefficient. Due to the task construction, it can be assumed that D-prime during the first learning time-point was equal to zero in all experimental conditions. This reduces the equation to the form:

(2) $$P_{n}\;=\;P_{\infty }-P_{\infty }e^{-An}$$

where *A* is a parameter corresponding to the shape of the curve and *P*_∞_is its horizontal asymptote. The first parameter can be interpreted as a general pattern of learning and the second one as the maximal level of accuracy possible to attain in a short time frame. The curve of this form was then fitted separately for every participant to find *P*_∞_ and *A*, which were then compared between conditions.

Finally, the literature focusing on behavioral analysis of the modulators of the learning process points to the role of not only reward or punishment itself, but the discrepancy between the predicted and received amount of reward (Garrison et al., 2013). The learning process continues until the difference—the PE—reaches a point of zero-value, i.e., the state where there is no longer any discrepancy between the predicted and obtained outcome (Schultz, 2016). The decrease in this discrepancy depends on the learning rate, i.e., how quickly a participant gathers the information necessary to minimize PE, and depends on multiple factors, such as reliability of feedback and the amount of information useful for the learning process associated with the particular stimulus (Ullsperger et al., 2014). Following Frank et al. (2007b) and Cavanagh et al. (2010), we use the modified Q-learning approach with different learning rate parameters corresponding to different sources of information during the task in order to assess the learning rate associated with different motivational conditions introduced in the PADL. Usually, in the Q-learning approach different *Q*-values are assigned to all possible reactions for a given stimulus and then updated each time the reaction occurs. However, in the PADL average number of actions which are never chosen oscillates around 20% of total possible actions (i.e., some distractor stimuli are never chosen and some targets are never rejected). Thus, the *Q*-values of never chosen stimuli remain at their initial level and in consequence, they disturb optimal model fitting. To resolve this issue, we have chosen simpler model in which the single *Q*-value is assigned to every stimulus as an estimate of the answer certainty, i.e., the 0 value corresponds to absolute certainty of stimuli being of different type that it actually is (i.e., being target for distractor stimuli and being distractor for target stimuli) and 1 to certainty of stimuli being of a correct type. The estimates are updated after each reaction for the stimulus and the feedback (or lack thereof) and initialized as 0.25, which corresponds to the probability that the stimulus may be a target and is not based on participants’ prior knowledge.

Four different models based on these premises were fitted separately for each participant and condition (i.e., each model was fitted 4^∗^58 = 232 times) using maximum likelihood estimation, where the mean of squared differences between *Q*-values and actual responses was chosen as the measure of error. To choose which of the four models the most accurately describes the learning process, they were compared using the Akaike Information Criterion (AIC, Akaike, 1974), using equation:

(3) AIC =−2ln(L)+2k

where *L* is a likelihood function and *k* is a number of model parameters.

The likelihood of every model was calculated for each participant as the probability of executed response, where the *Q*-values were interpreted as probabilities of choosing correct answers.

(1) Model 1 fitted single learning rate α for all types of stimuli and feedbacks according to the formula:

(4) $$Q_{s}(t+1)\;=\;Q_{s}(t)\;+\;\alpha [1-Q_{s}(t)]$$

where *r(t)* = 1 for correct and 0 for incorrect answer.
(2) Model 2 fitted separate learning rates for target and distractor stimuli both based on the same formula:

(5) $$Q_{s}(t+1)\;=\;Q_{s}(t)\;+\;\alpha_{T}[1-Q_{s}(t)]$$

(3) Model 3 fitted separate learning rates for correct and incorrect answers, based on formulas:

(6) $$Q_{s}(t\;+\;1)\;=\;Q_{s}(t)\;+\;(r\alpha_{+}\;+\;(1-r)\alpha_{-})[1-Q_{s}(t)]$$

for target stimuli and

(7) $$Q_{s}(t\;+\;1)\;=\;Q_{s}(t)\;+\;((r-1)\alpha_{+}\;+\;r\alpha_{-})[1-Q_{s}(t)]$$

for distractor stimuli, where *r* is participant’s response (1 – choose or 0 – avoid) for stimulus s. Note that only two parameters, namely α_+_ and α_-_, are fitted here.
(4) Model 4 fitted separate learning rates for correct and incorrect answers and target and distractor stimuli, therefore it uses information gathered from four sources: correctly chosen targets (*T*+), correctly rejected distractors (*D*+), incorrectly rejected targets (*T-*), and incorrectly chosen distractors (*D-*) based on the formulas:

(8) $${\text{Q}}_{\text{s}}{\text{(t + 1) = Q}}_{\text{s}}{\text{(t) + (rα}}_{\text{T+}}{\text{ + (1-r)α}}_{\text{T-}}{\text{)[1-Q}}_{\text{s}}\text{(t)]}$$

for target stimuli and

(9) $$Q_{s}(t\;+\;1)=Q_{s}(t)\;+\;((r-1)\alpha_{D+}\;+\;r\alpha_{D-})[1-Q_{s}(t)]$$

for distractor stimuli.

Note that in all the above models *t* refers to trial number, α is learning rate, and *s* denotes stimulus index.

The average AIC values over all model fits for models 1, 2, 3, and 4 equaled 35.4, 37.3, 35.2, and 33.6, respectively, hence model 4 was chosen for further analysis.

All the statistical analyses were performed using Statistica 12 (Stat-Soft Inc.) and Matlab 2015b (Mathworks, Inc.).

## Results

### Indicator of Task-Mastering

**FIGURE 2:**
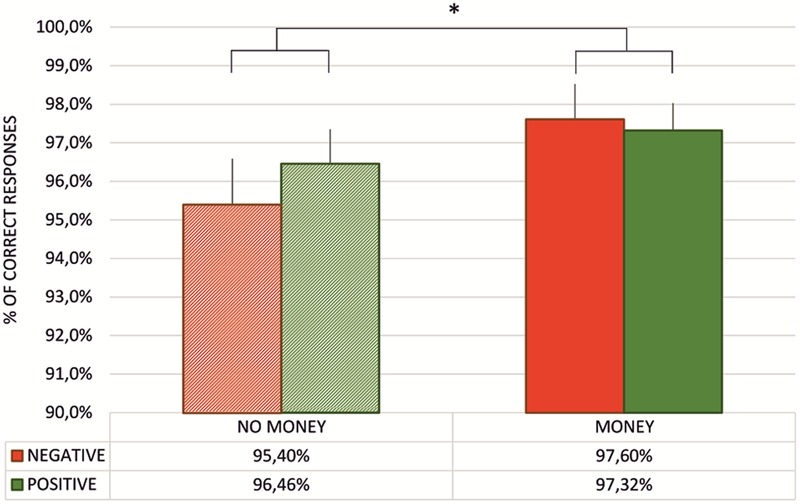
Mean percentage of correct responses in the test phase for every task block. The table presents the mean percentage of correct responses in subsequent test phases. Vertical bars denote standard errors. The asterisk denotes significant difference (^∗^*p* < 0.01).

To assess whether participants mastered the task, i.e., they learned the correct pairs, we counted the percentage of correct responses in the test phase after every single block. As presented in **Figure 2**, the percentage of correct responses was greater than 95% in all four cases. These results suggest that participants gathered all the necessary knowledge through the learning process and, as a consequence, responded with close to perfect accuracy. Moreover, the percentage of correct responses was submitted to a 2 × 2 repeated measures ANOVA with type of feedback (two levels: negative vs. positive feedback) and monetary incentive (two levels: no-money vs. money condition) factors. We observed a main effect of money [*F*_(1,57)_ = 9.12, *p* < 0.01, $\eta_{p}^{2}$ = 0.14] with the percentage of correct responses significantly higher in blocks where the performance score was associated with either gaining or losing money.

### Response Time

**FIGURE 3:**
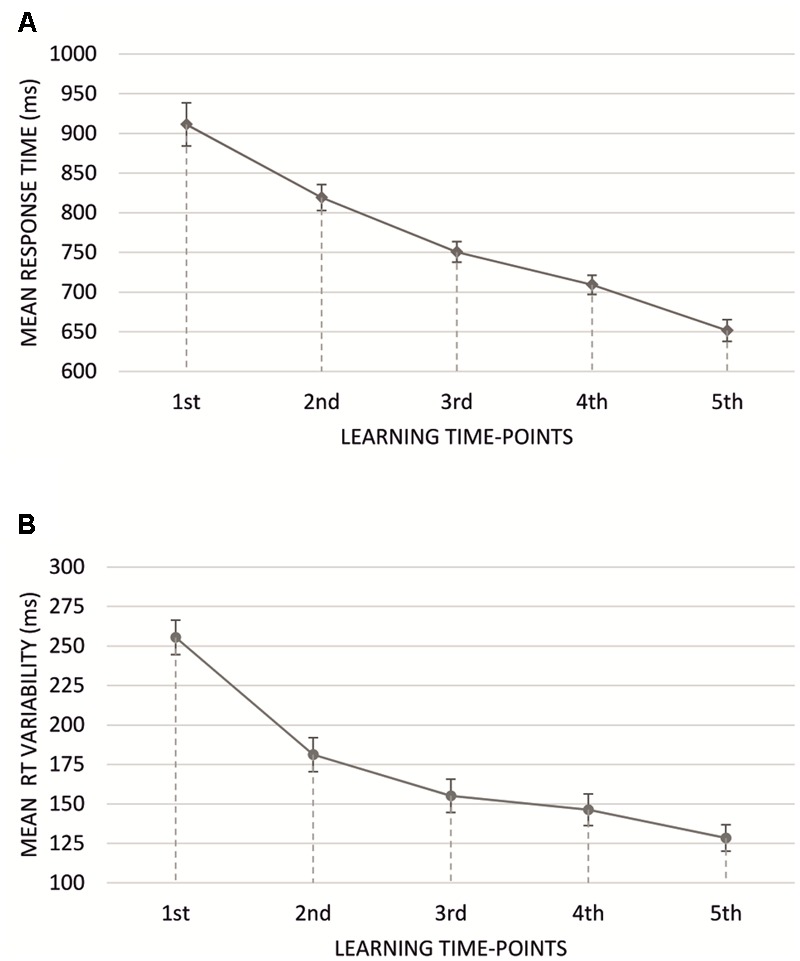
**(A)** Mean response times for subsequent learning time-points for all learning blocks. **(B)** Mean response time variability for subsequent learning time-points for all learning blocks. Vertical bars denote standard errors.

Mean RTs were submitted to a 2 × 2 × 2 × 5 repeated measures ANOVA with response accuracy (two levels: erroneous vs. correct response), type o feedback (two levels: negative vs. positive feedback), monetary incentive (two levels: no-money vs. money condition), and progress in learning (five levels: five learning time-points) factors (mean RTs are presented in **Table 1**). We observed a main effect of learning time-points [*F*_(4,228)_= 107.31, *p* < 10^-6^, $\eta_{p}^{2}$ = 0.65], with responses significantly faster in every subsequent learning time-point (**Figure 3A**). Main effects of neither response accuracy [*F*_(1,57)_= 0.58, *p* > 0.1], type of feedback [*F*_(1,57)_ = 0.19, *p* > 0.1] nor monetary incentive condition [*F*_(1,57)_ = 1.02, *p* > 0.1] were found.

**Table 1:** Mean RTs (in ms) for correct and erroneous responses for every task block (bad, good, loose, win) in subsequent learning time-points.

	Erroneous responses			
	*Bad*	*Good*	*Lose*	*Win*
**Learning time-point**				
1	935.59 (246.81)	812.60 (204.76)	906.64 (222.78)	937.57 (273.02)
2	846.46 (212.35)	841.74 (169.90)	835.00 (185.05)	836.26 (222.39)
3	744.55 (137.80)	793.47 (137.16)	772.97 (159.00)	783.24 (216.91)
4	755.51 (237.05)	738.68 (156.78)	694.79 (167.95)	697.91 (202.93)
5	606.54 (137.81)	680.43 (174.57)	636.58 (199.15)	578.81 (236.06)

	**Correct responses**			

**Learning time-point**				
1	970.11 (248.95)	836.25 (194.94)	911.43 (214.75)	947.38 (265.44)
2	821.70 (136.00)	814.62 (132.33)	802.44 (137.12)	809.65 (151.05)
3	749.98 (110.53)	767.33 (104.01)	727.22 (109.43)	748.00 (131.48)
4	721.75 (114.00)	710.17 (92.20)	695.94 (96.63)	710.09 (113.33)
5	661.55 (122.86)	653.28 (120.5)	642.43 (102.43)	653.43 (117.07)

*Standard deviations are presented in brackets.*

Further, we analyzed the dynamics of vRT using an ANOVA test analogical to that applied to the mean RT measure (**Table 2**). The analysis showed significant response accuracy, monetary incentive and progress in learning effect. The vRT was significantly lower for erroneous compared to correct responses [*F*_(1,57)_ = 27.60, *p* < 0.001, $\eta_{p}^{2}$ = 0.33] and blocks where there was a possibility of losing or winning money [*F*_(1,57)_= 8.19, *p* < 0.01, $\eta_{p}^{2}$ = 0.13]. The significant interaction of response accuracy and monetary incentive [*F*_(1,57)_= 5.58, *p* < 0.05, $\eta_{p}^{2}$ = 0.09] revealed significantly lower vRTs for erroneous responses in blocks with monetary incentive, compared to no money blocks (*p* < 0.01), whereas the vRT for correct responses did not differ due to the presence of monetary incentive. Moreover, vRT gradually decreased through the learning process [*F*_(4,228)_= 93.42, *p* < 0.001, $\eta_{p}^{2}$ = 0.62], being highest for the first learning time-point, and lowest for the fifth learning time-point (**Figure 3B**). The type of presented feedback did not significantly influence the magnitude of vRT [*F*_(1,57)_= 0.05, *p* > 0.5]. Additionally, we uncovered a significant interaction effect of response accuracy and progress in learning [*F*_(4,228)_ = 6.59, *p* < 0.001, $\eta_{p}^{2}$ = 0.10] and feedback type and progress in learning [*F*_(4,228)_= 4.09, *p* < 0.01, $\eta_{p}^{2}$ = 0.07] with vRT decreasing faster when participants committed errors (compared to correct responses), and when they were presented with negative feedback (compared to positive). The remaining interaction effects did not exceed the level of statistical significance.

**Table 2:** Mean RT variability (in ms) for correct and erroneous responses, for every task block (bad, good, loose, win) for subsequent learning time-points.

	Erroneous responses			
	*Bad*	*Good*	*Lose*	*Win*
**Learning time-point**				
1	275.88 (126.36)	233.06 (01.31)	272.95 (120.68)	255.26 (104.14)
2	182.23 (152.48)	176.55 (79.19)	170.57 (137.21)	165.31 (121.78)
3	140.23 (124.42)	163.57 (123.56)	114.08 (120.17)	131.20 (165.31)
4	148.54 (146.60)	159.81 (137.86)	93.03 (122.17)	123.75 (180.09)
5	104.29 (101.59)	131.07 (95.06)	85.89 (22.65)	95.61 (133.73)

	**Correct responses**			

**Learning time-point**				
1	280.04 (102.76)	231.99 (97.39)	245.04 (113.05)	249.45 (128.80)
2	206.74 (110.96)	174.86 (72.03)	188.64 (72.58)	184.13 (106.64)
3	175.77 (87.75)	174.21 (88.30)	168.35 (88.17)	174.00 (114.32)
4	172.13 (88.01)	163.51 (84.50)	147.06 (60.68)	163.09 (87.64)
5	150.34 (76.10)	149.03 (77.29)	155.00 (77.01)	156.48 (85.68)

*Standard deviations are presented in brackets.*

### Learning Dynamics

**FIGURE 4:**
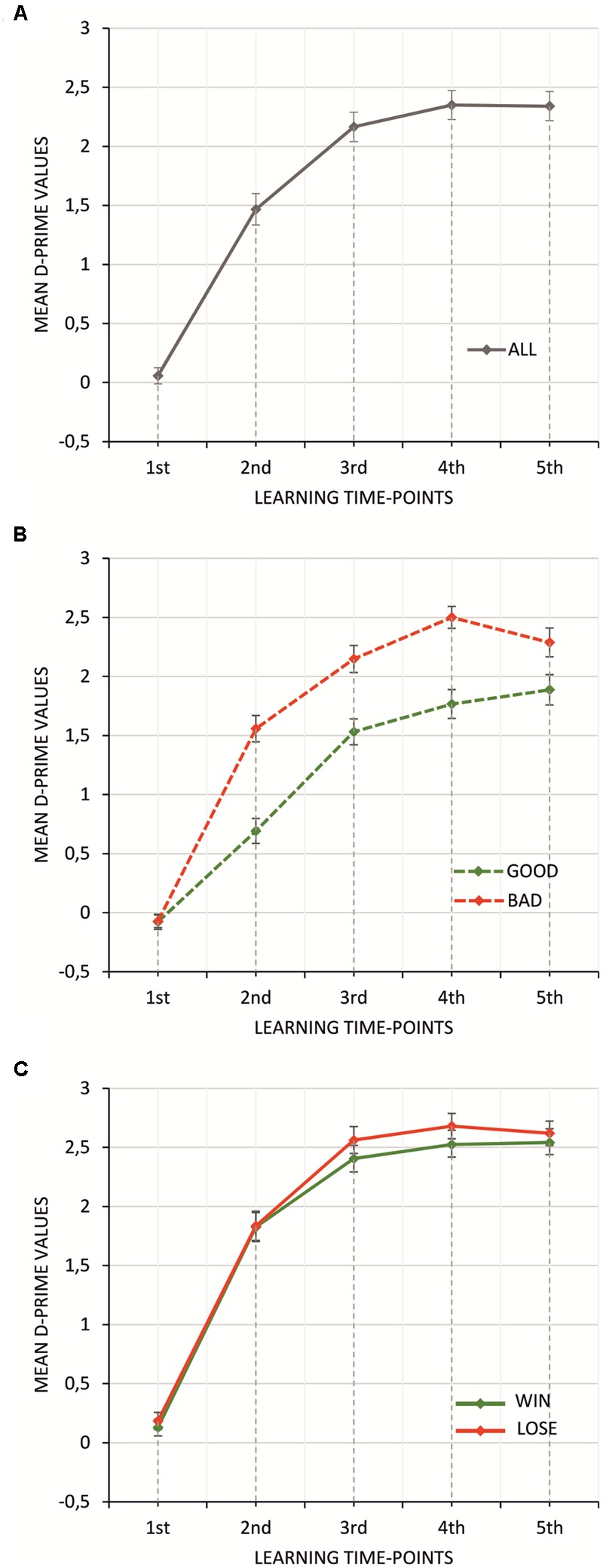
Mean D-prime values averaged separately for each trial in all **(A)**, good and bad **(B)**, and win and lose **(C)** conditions. Vertical bars denote standard errors.

**Figure 4** shows averaged participant performance in all trials for all experimental conditions.

**FIGURE 5:**
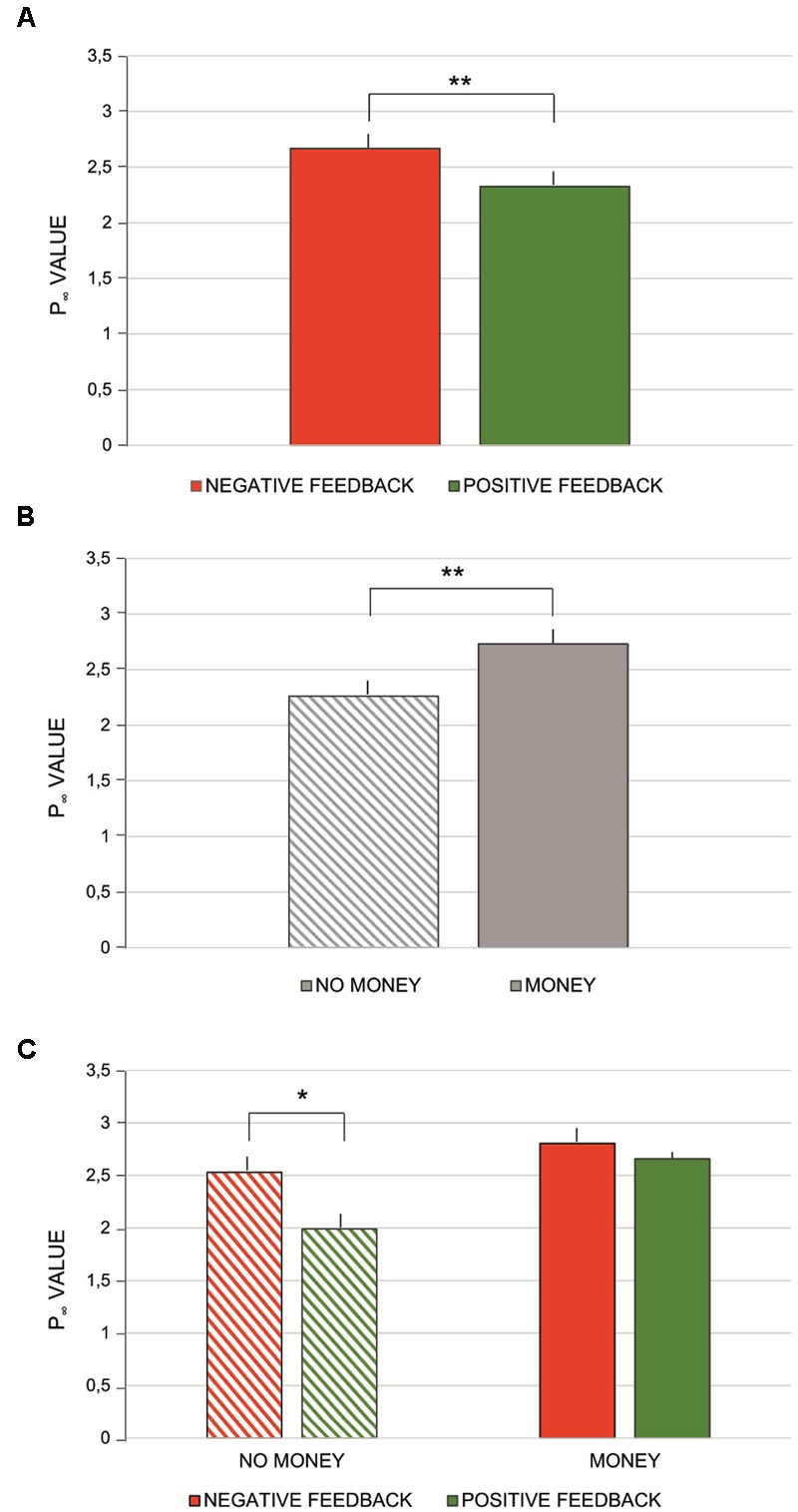
Mean *P*_∞_values computed for: **(A)** negative vs. positive feedback condition; **(B)** no-money vs. money condition; **(C)** interaction effect between money vs. money and negative vs. positive feedback conditions. Vertical bars denote standard errors. Asterisks denote significant differences (^∗^*p* < 0.0015; ^∗∗^*p* < 0.0001).

Both parameters of the learning curve model, *P*_∞_ and *A* (**Table 3**) were subjected to 2 × 2 repeated measures ANOVA with feedback type (two levels: negative vs. positive feedback) and monetary incentive (two levels: no-money vs. money condition) factors. No significant effect was found for parameter *A* [monetary incentive: *F*_(1,57)_= 0.43, *p* > 0.5; feedback type: *F*_(1,57)_ = 0.29, *p* > 0.5]. For *P*_∞_, the main effect of feedback type was observed [*F*_(1,57)_ = 17.03, *p* < 0.0001, $\eta_{p}^{2}$ = 0.23], indicating that the asymptote *P*_∞_ was significantly greater for negative than for positive feedback (see **Figure 5A**). The asymptote was significantly greater for conditions with financial reward [*F*_(1,57)_= 53.35, *p* < 0.0001, $\eta_{p}^{2}$ = 0.48] (see **Figure 5B**). The interaction effect of feedback type and monetary incentive on the asymptote was also significant [*F*_(1,57)_= 11.51, *p* < 0.01, $\eta_{p}^{2}$ = 0.17]. The Bonferroni *post hoc* test revealed a significant difference between blocks with negative and positive feedback in the no-money condition (*p* < 0.0001), while there was no significant difference in the money condition (*p* > 0.1) (see **Figure 5C**).

**Table 3:** Mean α and *P*_∞_ parameters for every task block.

	α	*P*_∞_
Bad	5.82 (11.77)	2.53 (0.83)
Good	5.56 (12.29)	1.99 (0.98)
Lose	7.56 (13.48)	2.80 (0.77)
Win	5.91 (11.68)	2.66 (0.77)

### Learning Rate Estimation

**FIGURE 6:**
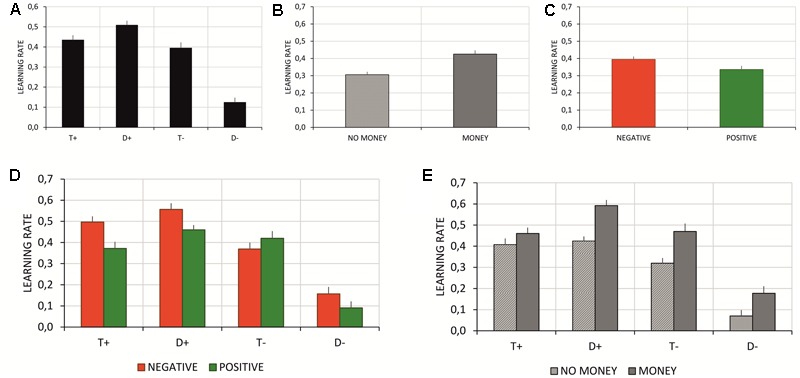
**(A)** Mean learning rates for all sources of information (T+, D+, T–, D–); **(B)** Mean learning rates for blocks with and without monetary incentive; **(C)** Mean learning rates for blocks with negative and positive feedback; **(D)** Mean learning rates all sources of information (T+, D+, T–, D–) in blocks with negative and positive feedback; **(E)** Mean learning rates all sources of information (T+, D+, T–, D–) in blocks with and without monetary incentive. Vertical bars denote standard errors.

Estimated learning rates (alphas) (**Table 4**) were subject to a 4 × 2 × 2 repeated measures ANOVA with information source (four levels: T+, T-, D+, D-), feedback type (two levels: negative vs. positive feedback), and monetary incentive (two levels: no money vs. money condition) factors. The *post hoc* analyses were performed using the Tukey HSD test. The analysis showed the main effects of information source and monetary incentive. Learning rates significantly differed with respect to information source [*F*_(3,171)_ = 74.14, *p* < 0.001, $\eta_{p}^{2}$ = 0.57]. The *post hoc* analysis showed that participants had the highest learning rate based on correctly chosen distractor stimuli, and significantly the lowest learning rate based on the stimuli incorrectly classified as distractors (see **Figure 6A**). The main effect of monetary incentive (**Figure 6B**) revealed learning rates significantly higher in blocks where the monetary incentive was introduced [*F*_(1,57)_ = 19.93, *p* < 0.001, $\eta_{p}^{2}$ = 0.26], and the main effect of feedback type (**Figure 6C**) revealed learning rates significantly higher in blocks with negative feedback [*F*_(1,57)_ = 11.03, *p* < 0.002, $\eta_{p}^{2}$ = 0.16]. The interaction analysis showed significant interactions between information source and feedback type [*F*_(3,171)_ = 6.75, *p* < 0.001, $\eta_{p}^{2}$ = 0.11] and between information source and monetary incentive [*F*_(3,171)_ = 4.39, *p* < 0.01, $\eta_{p}^{2}$ = 0.07]. Based on the *post hoc* test, compared to the no money condition, participants had a higher learning rate in blocks with a monetary incentive for all information sources, except the correctly classified targets (*p* = 0.7, see **Figure 6E**). Further, compared to blocks with positive feedback, the *post hoc* analysis of information source and feedback type interaction disclosed significantly higher learning rates for blocks with negative feedback when participants learned using information about correctly chosen targets (see **Figure 6D**).

**Table 4:** Mean learning rates for all the sources of information (T+, D+, T-, D-), for every task block.

	T+	D+	T-	D-
Bad	0.47 (0.27)	0.51 (0.32)	0.29 (0.03)	0.13 (0.05)
Good	0.34 (0.04)	0.34 (0.45)	0.35 (0.04)	0.01 (0.04)
Lose	0.52 (0.04)	0.61 (0.48)	0.45 (0.04)	0.19 (0.05)
Win	0.40 (0.05)	0.58 (0.57)	0.49 (0.04)	0.17 (0.05)

*Standard deviations are presented in brackets.*

## Discussion

The purpose of the present study was to test whether the newly design PADL task allows advancements in the deterministic learning process in varying motivational conditions to be described. Introducing a new, behaviorally verified task operating on the rules of deterministic learning provides an opportunity to depict learning dynamics using a relatively simple contingency learning model. This is especially important if we consider that groups with cognitive impairments do not always comply with the demands of commonly used probabilistic learning tasks, such as older adults (van de Vijver et al., 2014) or people with neurological or psychiatric disorders (Paus et al., 2001; Frank et al., 2004; Vandenberghe et al., 2006; McGirr et al., 2012; Endrass et al., 2013).

All the applied measures consistently show progress in individual performance. D-prime values, which constitute learning curves, depict increasing response accuracy. Decreasing response times and RT variability indicate increasing mastery of tasks, as in every subsequent learning time-point the response times were significantly shorter and more consistent than in previous ones. Overall, our results show that participants did learn while performing the PADL and prove it to be suitable for assessing changes during the learning process.

The analysis of learning curve dynamics and response time measures show that learning process dynamics are modulated by motivational conditions introduced in the task. In blocks where participants were presented with the possibility of losing or gaining money, they learned significantly quicker than in blocks with no monetary incentive, as indicated by the significant difference in the learning curve asymptote. The motivation to learn precedes the process of learning. Learning can be driven by anticipation of external or internal reward, such as good grades at school or self-satisfaction. The amount of effort we put into the learning process depends on the perceived value of the outcome (Adcock et al., 2006). Overall, as suggested by multiple theoretical accounts, introducing monetary incentives affects task performance by inducing increased effort to maximize the possible rewarding outcome, which leads to improvements in performance (Bonner and Sprinkle, 2002). The presented results are even more interesting when the delayed nature of payoff of considered. Participants learned faster even though the reward was associated not with the learning process itself, but with the recollection of learned pairs. The effect of monetary incentive was also observed when assessing the level of task mastery. Participants learned significantly more pairs in blocks followed by the test phase, in which there was the chance to either gain or not lose money for matching pictures correctly. Together, these results support the notion that introducing money as a motivator facilitates both the speed of the learning process and the number of memorized correct pairs.

Money was not the only factor affecting the learning performance. We compared the learning dynamics between blocks varying in provided feedback information. The results show the main effect of type of feedback information (see **Figure 3B**). In blocks where participants were presented with feedback after incorrect responses, the learning process was faster than in blocks with feedback about correct responses. As indicated by the exponential learning curve, the increase in the acquisition of knowledge about correct and incorrect pairs was sharper in the negative feedback condition. It has been shown that learners prefer to receive feedback after a “good” rather than a “poor” trial (Chiviacowsky and Wulf, 2007). Negative feedback after an erroneous response indicates an insufficient level of knowledge or skills required in the given task. Therefore, it is considered an irritating reminder that there is a need to change behavior and improve the learning process (Ullsperger and von Cramon, 2004). As noted by Bak and Chialvo (2001), the primary focus of the human brain is to get rid of irritating negative feedback signals. Additionally, from the evolutionary perspective, errors are treated as events that may place an organism in danger, therefore they should be avoided (Hajcak and Foti, 2008). In the light of the presented facts, it is not surprising that the process of learning was amplified in blocks where participants were presented only with negative feedback. The motivation to avoid information about “not being good enough” exceeded the motivation to learn in blocks where feedback was associated only with good responses. These results are also supported by the framing effect that derives from the classic prospect theory of loss aversion by Tversky and Kahneman (1981), which shows that loss is motivationally more significant than the equivalent gain. It should be noted that some studies report equal learning results from negative and positive feedback in healthy participants (Frank et al., 2004; Wheeler and Fellows, 2008). However, these studies operate on a concept of learning as an effect of the learning process, while we focus on learning dynamics: the way knowledge is gathered. Moreover, we also report no significant difference between blocks with positive feedback and blocks with negative feedback in the percentage of correct responses in the test phase, which is an indicator of task mastery.

Finally, we found the interaction effect of feedback type and monetary incentive factors. A significant effect of feedback type was revealed for blocks without any monetary incentive, while there was no significant effect for blocks in which participants were informed about the possibility of losing or gaining money. At first, these results seem surprising: how does receiving negative rather than positive feedback without the prospect of a payoff lead to increased learning speed, and how is a similar effect absent when we introduce the possibility of losing or winning money? As mentioned before, the perceived value of the outcome determines the amount of effort we put into the learning process. In blocks with money incentives, participants could win a total of 30 PLN (∼8$): they had to pair all the stimuli correctly in the test phase to either win the full amount (15 PLN, ∼4$), or not lose anything from the given sum (15 PLN, ∼4$). Thereby, the desired effect in both blocks was the same: to get as much money as possible. In consequence, the motivational value of both monetary conditions was similar and affected participants’ performance in the same manner. When participants know that they can win money, the type of presented feedback does not seem to make a difference. Further, it can be concluded that the prospect of a monetary incentive overrides the influence of the aversive effect of negative feedback, as described by Tversky and Kahneman (1981). This result becomes less surprising when we consider the scale of reference on which we put gains and losses. When we assume the scale is bipolar, meaning we compare something considered as “good” with something considered as “bad,” the loss aversion mechanism almost always works (see Novemsky and Kahneman, 2005). However, when the difference between loss and gain is not so easily interpreted, the loss aversion mechanism may not act as classic prospect theory suggests (McGraw et al., 2010). In our experiment, participants’ perception of the difference between winning and losing money may be based on the simple assumption that they must learn equally effectively in both the winning and losing conditions to minimize the risk of not receiving the full amount of money provided for the task.

It should be noted that the design of the PADL involves a fixed order of blocks with purely cognitive feedback always preceding the possible reward. Despite the observed difference in the learning process between conditions, the block order may act as the interfering factor. To exclude the possibility of practice effect accounting for differences between the blocks with and without monetary incentive, a follow-up study with a mixed condition needs to be conducted. Although we believe the use of a fixed order was justified, it is also a design limitation.

The literature focusing on behavioral analysis of the modulators of the learning process indicates the role of not only reward or punishment, but the discrepancy between the predicted and received amount of reward (Garrison et al., 2013). The learning process continues until the difference—the PE—reaches a point of zero-value, i.e., the state where there is no longer any discrepancy between the predicted and obtained outcome (Sutton and Barto, 1998; Hare et al., 2008; Schultz, 2016). The decrease in PE value depends on the speed of an individual’s learning process, i.e., the learning rate. Introducing the Q-learning model as a method to assess trends in learning makes it possible to investigate the differences in learning due to the source of utilized information and provides an opportunity to more thoroughly test whether the experimental paradigm we have introduced allows us to study the learning process (Schultz and Dickinson, 2000; Cavanagh et al., 2010; Den Ouden et al., 2012). According to the results we obtained using our modified Q-learning model, participants presented the highest learning rate when they based learning on information from correctly chosen distractor stimuli. On the other hand, the least learning-beneficial information, which is associated with correctly rejected distractors, resulted in the lowest observable learning rate. Moreover, similar to the dynamics of the learning curve, the learning rate was sensitive to motivational manipulation. When learning occurred in blocks without monetary incentive, the learning rate was significantly lower than in blocks with the possibility of receiving money (see **Figures 6B,E**), except the correctly chosen targets, which were treated by participants as redundant information. Again, this indicates the highly motivational value of monetary incentives, even when postponed.

Additional support for the described modulatory effect of the motivational conditions introduced in the PADL comes from the analysis of RT variability. Even though the change in RT itself was influenced only by progress in the learning process, its variability across the learning time points was sensitive to task conditions. The homogeneous RTs across learning conditions rule out the possibility that participants manipulated the learning strategies they used depending on the experimental block and exclude the possibility that different learning strategies account for the differences in RT variability (Toni et al., 2001). Studies reporting differences in RT variability indicate a strong relationship between this performance measure and executive control of performed actions. For example, lapses of attention in older adults result in increased RT variability (Myerson et al., 2007). Moreover, children diagnosed with ADHD, who are known to have impaired ability to control the focus of attention, can also be characterized by increased RT variability (Epstein et al., 2011). Considering this data, the decrease in response time variability may be interpreted as a result of the opposite process: an increase of attention engagement in the task. In addition, the significantly smaller RT variability in blocks in which participants were either presented with the prospect of a monetary incentive or were presented with negative feedback indicates more profound cognitive engagement in these parts of the task due to their motivational properties.

To summarize, our study presents the dynamics of the learning process using a newly designed deterministic learning task, the PADL. All the measures implemented to assess the learning process (i.e., level of task mastery, RT analysis, RT variability analysis, learning curve dynamics, and learning rate) clearly show that the PADL is an experimental paradigm that is well-suited to studying learning in a deterministic environment. We prove the PADL to be a useful tool while assessing condition-induced differences during learning. Particularly, we show that the learning process itself is influenced by manipulating both the type of feedback information and the motivational significance associated with the expected monetary reward.

## Author Contributions

MG, EB, AD, AG, TM, and JM-K: Substantial contributions to the conception or design of the work; or the acquisition, analysis, or interpretation of data for the work; Drafting the work or revising it critically for important intellectual content; Final approval of the version to be published; Agreement to be accountable for all aspects of the work in ensuring that questions related to the accuracy or integrity of any part of the work are appropriately investigated and resolved.

## Conflict of Interest Statement

The authors declare that the research was conducted in the absence of any commercial or financial relationships that could be construed as a potential conflict of interest.
